# *Drosophila* Lysophospholipase Gene *swiss cheese* Is Required for Survival and Reproduction

**DOI:** 10.3390/insects13010014

**Published:** 2021-12-22

**Authors:** Pavel A. Melentev, Eduard G. Sharapenkov, Nina V. Surina, Ekaterina A. Ivanova, Elena V. Ryabova, Svetlana V. Sarantseva

**Affiliations:** Petersburg Nuclear Physics Institute Named by B.P.Konstantinov of National Research Center «Kurchatov Institute», 188300 Gatchina, Russia; melentev_pa@pnpi.nrcki.ru (P.A.M.); e_sharapenkov@mail.ru (E.G.S.); surina_nv@pnpi.nrcki.ru (N.V.S.); ivanova_ea@pnpi.nrcki.ru (E.A.I.); ryabova_ev@pnpi.nrcki.ru (E.V.R.)

**Keywords:** *swiss cheese*, lysophospholipase, *Drosophila melanogaster*, fertility, testis, lipid droplets, spermatozoa, *PNPLA6*, *NTE*, cyst cells

## Abstract

**Simple Summary:**

Biological evolution implies fitness of newly evolved organisms that have inherent adaptive traits because of mutations in genes. However, most mutations are detrimental, and they spoil the organism’s life, its survival and its ability to leave progeny. Some genes are extremely vital for an organism, and therefore, they tend to save their structure and do not mutate or do it very composedly. That is the case of the gene encoding PNPLA6 lysophospholipase domain that evolved in bacteria, and evolution obliged it to save its function in higher animals. In mammals, complete dysfunction of such a gene is lethal because of its high importance in placenta for early embryo development. Why is it conserved in other species, for instance insects, that have no placenta? Here we studied the role of the PNPLA6-encoding gene named *swiss cheese* in *Drosophila melanogaster* fitness. We have found that its dysfunction results in premature death of specimens and their inability to leave enough progeny. Thus, we provide the first evidence for significance of the gene that encodes the lysophospholipase enzyme in fitness of insects.

**Abstract:**

*Drosophila melanogaster* is one of the most famous insects in biological research. It is widely used to analyse functions of different genes. The phosphatidylcholine lysophospholipase gene *swiss cheese* was initially shown to be important in the fruit fly nervous system. However, the role of this gene in non-nervous cell types has not been elucidated yet, and the evolutional explanation for the conservation of its function remains elusive. In this study, we analyse expression pattern and some aspects of the role of the *swiss cheese* gene in the fitness of *Drosophila melanogaster*. We describe the spatiotemporal expression of *swiss cheese* throughout the fly development and analyse the survival and productivity of *swiss cheese* mutants. We found *swiss cheese* to be expressed in salivary glands, midgut, Malpighian tubes, adipocytes, and male reproductive system. Dysfunction of *swiss cheese* results in severe pupae and imago lethality and decline of fertility, which is impressive in males. The latter is accompanied with abnormalities of male locomotor activity and courtship behaviour, accumulation of lipid droplets in testis cyst cells and decrease in spermatozoa motility. These results suggest that normal *swiss cheese* is important for *Drosophila melanogaster* fitness due to its necessity for both specimen survival and their reproductive success.

## 1. Introduction

PNPLA6-domain encoding genes are evolutionarily conserved through pro- and eucaryotes [1,2,3], and they control phosphatidylcholine lysophospholipase activity in cells [3,4,5,6,7,8]. The first evidence for the physiological role of such a gene was shown for the fruit fly gene named *swiss cheese* (*sws*) [9]. It was found to provide the morphological stability and survival of neurons and glia in *Drosophila melanogaster* [9,10,11,12,13]. Flies with *sws* dysfunction are born normal but then have progressive neurodegeneration in the brain, reduced lifespan, locomotor activity decline [9,13,14]. It is accompanied with phosphatidylcholine (PC) and lysoPC increase [8,10,15] and accumulation of lipid droplets [13]. 

Later it became evident that orthologues of *sws* have similar role in the nervous system of vertebrates [7,16,17,18,19]. Compound heterozygous or homozygous recessive mutations in human *sws* orthologue called *PNPLA6* induce neurological abnormalities, affecting spinal cord [19,20,21], cerebellum [22,23,24,25,26,27,28,29,30,31,32,33,34,35,36], pituitary gland [24,26,31,37,38] and photoreceptor neurons [33,36,38,39,40]. Furthermore, in mammals *PNPLA6* is maintained because of its vital role in placenta development, so knockouts are lethal [41,42].

Both SWS and PNPLA6 proteins have the evolutionarily conserved patatin-like phospholipase domain [1,2,43]. PNPLA6 was shown to act as a phospholipase B that preferentially cleaves PC at both A1 and A2 sites [4,5,6,7,8,9]. The most favourable substrate for this lipase is lysoPC, i.e., the monoacylated one [4,5]. Functional alterations of PNPLA6 due to the gene mutations or organophosphate-induced inhibition of the protein affect lysoPC level [5,15]. Taking all the above into consideration, it is proposed that similar activity is inherent for SWS.

At the same time, besides the neurological phenotypes, no other deviations in the organism that lack SWS/PNPLA6 activity were discovered, despite its transcripts were found in many tissues [44,45]. In this work, we are the first to describe the normal *sws* expression pattern, revealing it in male reproductive system, and analyse survival plus reproduction rates of specimens that are deficient for SWS activity, finding out that the studied gene is crucial for male fertility.

## 2. Materials and Methods

### 2.1. Drosophila Stocks and Feeding

For visualization of cells with the active *sws* promoter, we used GAL4/UAS system [46], where GAL4 was taken from the y[*],w[*],P{w[+mW.hs] = GawB}sws[NP4072]/FM7c stock (KYOTO DGGR N°104592, hereinafter abbreviated as *sws-GAL4*, kindly donated by Halyna Shcherbata) and a fluorescent reporter was taken from the *w; UAS-nlsLacZ, UAS-CD8::GFP* stock (BDSC N°5137, hereinafter abbreviated as *UAS-CD8-GFP*). In addition, *w[1118]; P{w[+mC] = UAS-RedStinger}6* stock was used to express fluorescent reporter in nuclei (BDSC №8547, hereinafter abbreviated as *UAS-NLS-DsRed*).

To analyse consequences of *sws* dysfunction, we studied *sws^1^* stock [32], kindly donated by Halyna Shcherbata. As controls, *OregonR* and *CantonS* lines were used.

For the RNAi-dependent *sws* knockdown, the *y^1^ v^1^; P{TRiP.HMJ23229}attP40* line was used (BDSC №61338, abbreviated in this article as *UAS-sws-RNAi*).

*P{GawB}elav^C155^* stock (BDSC №458, abbreviated in this article as *elav-GAL4*) was used for the GAL4 transcription activator synthesis in neurons, w[1118]; P{w[+m*] = GAL4}repo/TM3, Sb[1] stock (BDSC №7415, abbreviated in this article as *repo-GAL4*) was used to express GAL4 in glia, whereas *w; +; tub-Gal4/TM3,Sb* (abbreviated in this article as *tub-GAL4*; kindly donated by Halyna Shcherbata) was used for induction of expression of the GAL4 transcription activator in the all fly cells.

In all experiments, except the longevity assay, flies were kept at +25 °C on the standard food (35 g semolina, 40 g sucrose, 25 g dry yeast, 4 g agar, 7 mL propionic acid per 1 L of distilled water) for feeding and breeding. In the longevity assay, flies were kept on 2.2% agar with a 100 μL droplet of yeast suspension (2 g of dry yeast diluted in 10 mL of dH_2_O).

If the sex of species is not specified, we analysed only males.

This study was approved by the Ethical Committee of the Petersburg Nuclear Physics Institute named by B.P. Konstantinov of NRC “Kurchatov Institute” (protocol # 01/КПБ of 13 January 2020).

### 2.2. Dissection of Species for Confocal Microscopy

To analyse embryos, eggs were collected and moved to phosphate buffered saline (PBS), washed, and incubated then in 3% sodium hypochlorite for 3–5 min with consequent washing in PBS. Then freshly prepared 4% paraformaldehyde (PanReac AppliChem, Barcelona, Spain) was added for 20 min fixation, and final washing in PBS was applied (3 times, 10 min). Samples were transferred on a glass with a drop of halocarbon oil, covered with a coverslip and analysed, using the Leica LX laser confocal microscope (Leica microsystems, Wetzlar, Germany).

Larvae were dissected with entomological needles and Vannas Spring Scissors (Cutting Edge 3 mm, # 15000-10, Fine Science Tools, Heidelberg, Germany), and organs were removed with forceps. Similarly, imagoes of 1, 15, 30, 45, 60 and 75 day-olds were dissected. Then, organs were fixed in freshly prepared 4% paraformaldehyde (PanReac AppliChem, Barcelona, Spain) for 20 min and washed in PBS (3 times, 10 min). Finally, organs were placed in a drop of Antifade Mounting Medium (Vectashield, Vector Laboratories, Burlingame, CA, USA) on a glass. For visualization of cell nuclei, DAPI dye was added (1:1000, Sigma, Merck, Darmstadt, Germany). Series images (1 to 2-μm-thick) were obtained with the Leica LX laser confocal microscope (Leica microsystems, Wetzlar, Germany) using 10, 20 air and 40, 63 oil immersion objectives. Fluorescence excitation was induced with the 405 nm laser for DAPI visualisation and 488 nm for GFP visualisation. LeicaLASAF and LASX software was used for image processing.

### 2.3. Quantitative Analysis of mRNA Level

The total RNA was obtained from ovaries and testes of at least ten 5-day-old virgin specimens using QuickRNA™ Mini Prep (ZymoResearch, Irvine, CA, USA) according to the manufacturer’s protocol. Genomic DNA was depleted using 8 U DnaseI (ThermoScientific, Waltham, MA, USA) application directly to a column matrix (for this, 80 μL of a buffer was applied, eluted, incubated for 30 min at 37 °C and reloaded into a column). cDNA was synthesized from 10 μL of purified RNA by reverse transcription kit (SK021, Evrogen, Moscow, Russia) using 340 U MMLV-RT and both random6 (2 μM) and oligo-dT (1 μM) primers. Then, qPCR was performed using iTaq Universal SYBR Green Supermix (Bio-Rad, Hercules, CA, USA) in a total volume of 10 μL with 2 μL of obtained cDNA and 50 pM of each primer, using CFX96 thermocycler (Bio-Rad, Hercules, CA, USA). There were 50 cycles (96 °C—30 s, 50 °C—30 s, 72 °C—30 s) and subsequent melt-curve analysis for verifying the single product presence in each reaction. Primers were common for all known *sws* transcripts (5′ to 3′): ACTACTCAATCATCAAATCTCC and CAGGATTGTGGGTTAATCG. As a reference gene, *Gapdh2* was chosen, and the corresponding primers were the following (5′ to 3′): GATGAGGAGGTCGTTTCTAC and ACCAAGAGATCAGCTTCAC. Measured Cq values from 3 biological multiplied by 3 technical replicates were used to assess the relative *sws* transcripts level normalized to the *Gapdh2* mRNA level.

### 2.4. Analysis of F1 Generation Survival

For analysis of species survival before the imago stage, virgin males and females were collected for 24 h, then five males and five females were placed together in a vial for 48 h and then flipped to a new vial until the loss or death of the first fly, but no more than 10 days. After flipping parents out, the vials were incubated for 16 days and then phenotypes of the first-generation progeny were registered, and all flies, hatched and dead pupae, were counted.

For analysis of imago survival, one- to two-day-old males were placed in test tubes with agar and yeast suspension and were kept at +25 °C (30–40 males per vial, with a total of at least 200 males per genotype). Live flies were flipped to a new vial with a fresh medium every 2–3 days, and the number of dead individuals was counted. The experiment was conducted until the death of the last fly.

### 2.5. Fertility and Fecundity Analysis

Virgin males and females were collected for 24 h, then were incubated separately for 24 h, and finally were placed in pairs in individual vials with the standard food (described above) with one exception: the quantity of agar was 11 g per 1 L. The vials were incubated for 24 h before replacing the bottom part of the vial that contained food and laid eggs. The eggs were counted immediately, and the bottom was placed for 48 h incubation after which unhatched eggs were counted. The experiment was performed until at least the 10th day after the first bottom replacement.

### 2.6. Behaviour Analysis

Locomotor activity and negative gravity taxis were tested using the RING assay, as described in [47]. Briefly, six groups of 20–40 flies of each genotype and age were transferred into empty 50 mL falcons without anaesthesia, and the vials were loaded into the RING apparatus. The apparatus was rapped three times in rapid succession to initiate a negative geotaxis response. The flies’ movements in tubes were videotaped and digital images captured 3 s after initiating the behaviour. The distance between a fly and a vial bottom was calculated for each fly. The performance of flies was analysed in six consecutive trials (interspersed with a 60 s rest). For each genotype and age, more than 200 flies participated in the experiment, and for each fly, six replicate values were obtained. To equalize the total sample size, 2000 values were chosen randomly among the obtained data sample for every genotype and age.

Male sex behaviour was analysed according to [48]. Naive males were collected and put in vials separately, incubated for 4–6 days and then tested with the same fertilized 5-day-old *CantonS* female. For the test, a male was placed in the camera in a separate part for 45 s to adapt, and then a barrier between the male and the female was opened. For the next 300 s, different types of male behaviour were registered: locomotor activity, preening, rest and courtship (orientation and following, vibration, copulation attempts). The summary time spent for each behaviour type was registered, and the courtship index was determined as a ratio of the summary time of courtship to 300 s.

For analysis of the sperm undulation activity, 5–7-day-old males were dissected, and seminal vesicles were extracted and put in a drop of physiological saline, where they were slightly teared with a needle. The sample was moved on an objective table of a microscope with a video camera as described [49]. The video was taped for 20 s with a frame rate of 60 frames per second. The video files were analysed with the ImageJ software (in a manual tracking mode). The track for the distal part of a spermatozoid tail was generated, and instant speed was determined for each two neighbour frames. Finally, the average speed for the tail was counted. The sample size was 111 spermatozoa obtained from ten different males.

### 2.7. ATP Relative Level Determination

The ATP level was evaluated with the ATP determination kit (A22066, Molecular Probes, Invitrogen, Thermo Fisher Scientific, Waltham, MA, USA) according to [50], with modifications. Testes and seminal vesicles of five males were collected in PBS on ice and homogenized in lysis buffer (6 M guanidine HCl, 100 mM Tris (pH 7.8), 4 mM disodium EDTA). Then, the sample was boiled for 5 min, centrifuged (+10 °C, 14,100× *g* rcf, 3 min), and 10 μL was dissolved in 90 μL of dilution buffer (25 mM Tris (pH 7.8), 100 μM disodium EDTA) and centrifuged again (+10 °C, 14,100× *g* rcf, 3 min). Next, 10 μL of the sample was added to a well of a plate with 100 μL of the ATP determination reagent. The total luminescence was measured using the plate reader (EnSpire2300, Perkin Elmer, Waltham, MA, USA) five times for each replicate. The values obtained for the ATP levels were normalized to the protein concentration measured by the standard protocol of the Bradford assay [51]. The experiment was conducted in five biological replicates multiplied by four technical replicates, so that the total size was 100 values in the sample.

### 2.8. Lipid Droplet Visualization

At least 20 testes for every genotype and age were dissected in cold PBS, fixed in 4% solution of paraformaldehyde (PanReac AppliChem, Barcelona, Spain) for 15 min and rinsed in PBS for 10 min three times. For visualization of lipid droplets [52], we added BODIPY 493/503 (Invitrogen, Thermo Fisher Scientific, Eugene, OR, USA) diluted in PBS in the concentration of 3 mM for 30 min, followed by washing in PBS for 15 min three times. Then, the testes were placed in the Mounting Medium with DAPI (Abcam, Cambridge, UK) for the same-day imaging. Series of images (distance between images 1 μm) were obtained with the Leica LX confocal microscope (Leica microsystems, Wetzlar, Germany). Fluorescence excitation was induced with the 405 nm laser for DAPI visualisation and 488 nm for BODIPY visualisation. LeicaLASAF and LASX software was used for image processing.

### 2.9. Oxidative Particle Measurement

The reactive oxygen species (ROS) level was measured using 2′,7′-dichlorodihydrofluorescein diacetate (H2DCF-DA, Invitrogen, Thermo Fisher Scientific, Waltham, MA, USA), as described in [53]. Testes and seminal vesicles from five males for each biological replicate, among five total replicates, were collected in PBS on ice and homogenized in a buffer containing 100 μL of 10 mM Tris (pH 7.4) and 3 μL protease inhibitor (Roche, Basel, Switzerland). The homogenate was centrifuged (for 10 min at 10,000× *g* rcf, +10 °C). Then, 5 μL of clear supernatant was mixed with 60 μL of 5 μM H2DCF-DA and incubated for 60 min at +37 °C. The fluorescence emission of DCF resulting from H2DCF-DA oxidation and hydrolysis was scanned at 485 nm excitation and 530 nm emission with the plate reader (EnSpire2300, PerkinElmer, Waltham, MA, USA). The values obtained for the ROS levels were normalized to a protein concentration measured by the standard protocol of the Bradford assay. The experiment was conducted in five biological replicates and three technical replicates with three fluorescence measurements per each replicate, so that the total size was 45 values in the sample.

### 2.10. Results Processing

Statistical analysis was performed using KyPlot 5.0 software. All samples were tested for normality with the Shapiro–Wilk test. If the distribution was normal, we used parametric tests: the Student’s *t*-test for a comparison of two samples and Dunnett’s test for multiple comparisons (three or more samples). For normally distributed samples, data were presented as histograms (mean ± 95% confidence interval (CI)). For other distribution types, nonparametric tests were used. For a comparison of two samples, the Mann–Whitney test was applied, whereas for multiple comparisons (three or more samples), the Steel test was performed. For samples where the Shapiro–Wilk test suggested a non-normal distribution, data were presented as box-and-whisker plots. In all cases where there were several technical replicates, we did not average them but put all the values in one sample, assuming that each value is an independent representative of an entire assembly of a studied parameter. For statistical analysis of phenotypical distribution in F1 we used chi-square test. The threshold for *p*-value was chosen as 0.05.

## 3. Results

### 3.1. Pattern of sws Expression during Drosophila Melanogaster Ontogenesis

**Figure 1:**
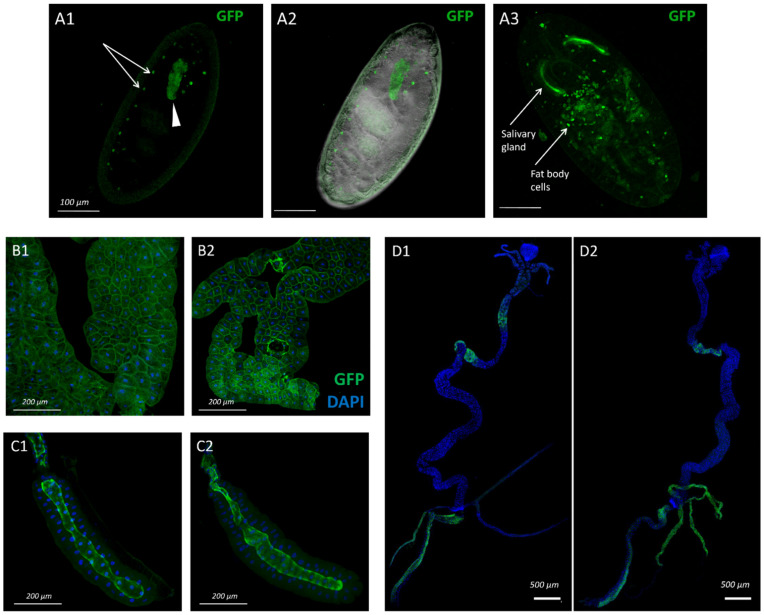
The cells and organs with GFP (green) expression in specimens with *sws-GAL4;UAS-CD8-GFP* genotype. (**A**) Embryo and (**B**–**D**) third instar larva. (**A1**,**A2**) Images of the embryo of the stage 16: (**A1**) confocal image showing GFP distribution, (**A2**) merged images (light + confocal) showing inner structure of the embryo and GFP. Metameric dots (presumably, according to sensilla) are marked with arrows, and the salivary gland is marked with the arrowhead. (**A3**) Stage 17 of embryo development; salivary glands and fat body cells are marked with arrows. (**B**) Fat body, (**C**) salivary gland, (**D**) intestine and Malpighian tubes of (**B1**,**C1**,**D1**) male and (**B2**,**C2**,**D2**) female. (**B**–**D**) Nuclei are stained with DAPI (blue). Scale bar: (**A**)—100 µm, (**B**,**C**)—200 μm, (**D**)—500 µm.

Transcriptomic data indicate that *sws* is expressed not only in the nervous system, but also in other tissues [44,45]. Therefore, we decided to describe the pattern of *sws* transcription activity during the ontogenesis of *Drosophila melanogaster*, with the exception of the nervous system, where its expression pattern had been defined [10,12]. To do this, we used the GAL4-carrying transgene with the *sws* promoter and detected its expression by virtue of the transmembrane fluorescent reporter synthesis in flies with *sws-GAL4;UAS-CD8-GFP* genotype. It let us visualize membranes of the cells which normally express *sws* in the *Drosophila melanogaster* organism during its development. CD8-GFP signal appearance firstly occurred in the embryo of the 16th developmental stage when the midgut was fully closed and gastric caeca were formed (Figure 1(A1,A2)). The signal was localized in salivary glands and metameric pairs of regions, presumably, referring to sensilla. Later, during the 17th stage, CD8-GFP started to accumulate throughout the whole embryo in the fat body cells (Figure 1(A3)). In the larvae of 1–3 instar, the fat body cells as well as the salivary gland cells remained CD8-GFP positive (Figure 1(B1,B2,C1,C2)). In addition, a less bright signal was detected in two regions of the intestine: in the midgut and in the hindgut. Malpighian tubes also showed reporter fluorescence (Figure 1(D1,D2)).

**Figure 2:**
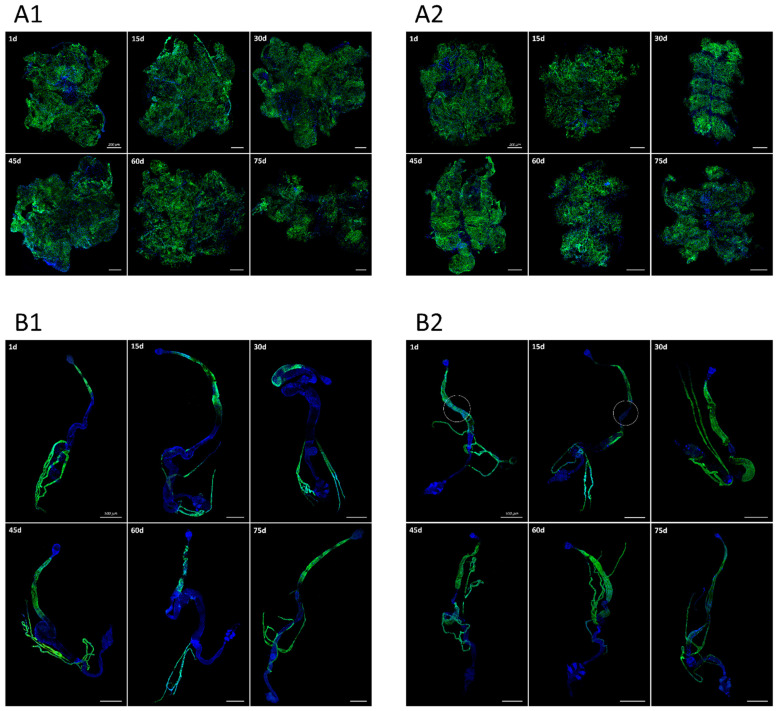
The cells with GFP (green) expression in the organs of imagoes with *sws-GAL4;UAS-CD8-GFP* genotype. (**A**) Fat body, (**B**) intestine and Malpighian tubes of (**A1**,**B1**) males and (**A2**,**B2**) females. Nuclei are stained with DAPI (blue). In the left upper corner of each photograph, the age of imago (days) is written. Scale bar: (**A**)—200 µm, (**B**)—500 µm.

We then examined the reporter expression in the *sws-GAL4;UAS-CD8-GFP* transgenic imago. We found a quite similar pattern of CD8-GFP distribution in males and females. The fluorescent signal was observed in the fat body throughout the first 75 days of adult life (older flies were not analysed since 75-day-old flies were considered to be aged enough to study *sws* expression upon aging). According to the pattern of signal distribution (Figure 2(A1,A2)), adipocytes but not oenocytes can express *sws*. Cells of the midgut and Malpighian tubes also showed the fluorescent reporter signal in males and females of all ages examined (Figure 2(B1,B2)). Even though the midgut of imago was CD8-GFP positive during the first 75 days of the fly life, the signal disappeared in the middle zone of the midgut in the males since approximately the 15th day. This morphological zone is associated with the activity of copper cells that performs acidification of the intestinal lumen, whereas the midgut itself is the main digestive region of the insect intestine [54]. These findings indicate that *sws* expression may be important for cells with a high demand for lipid turnover for endo- or exocytosis, as well as for lipid metabolism and storage.

**Figure 3:**
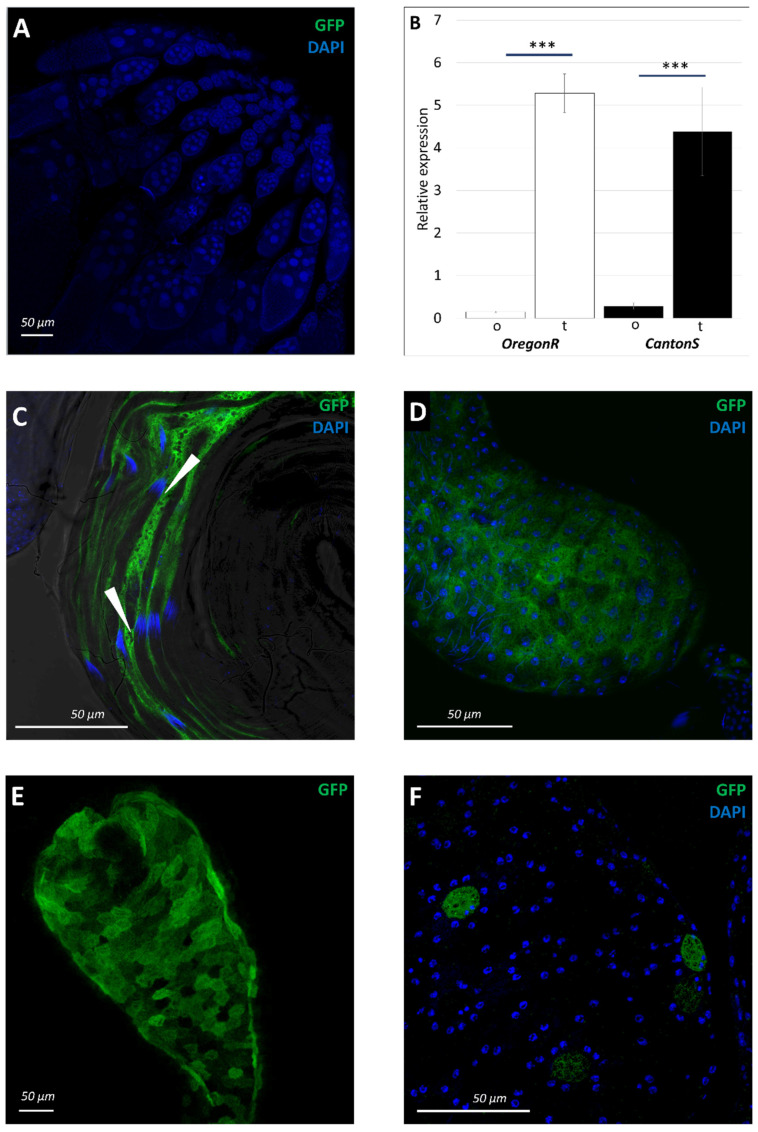
Analysis of *sws* expression in imago’s reproductive system. (**A**) Confocal image of the ovary of the female with *sws-GAL4;UAS-CD8-GFP* genotype. Nuclei are stained with DAPI (blue). (**B**) The relative level of *sws* transcripts in ovaries (o) and testes (t) of the wild type flies. The level of *Gapdh2* mRNA is accounted as 1 in each sample. Mean and 95% CI are shown; Student *t*-test; *** *p* < 0.001; *n* = 9. Note that there is significantly higher *sws* mRNA level in testes compared to those in ovaries (about 100 folds difference). (**C**–**F**) Cells with GFP (green) expression in the male reproductive system of 2–7-day-old imago with *sws-GAL4;UAS-CD8-GFP* genotype. Confocal images of (**C**) proximal part of the testis, (**D**) seminal vesicle, (**E**) anterior ejaculatory duct, (**F**) accessory gland. Nuclei are stained with DAPI (blue). Note that inside a cyst, there is no green signal, whereas along cysts there is (marked with arrowheads in box (**C**)). (**A**,**C**–**F**) Scale bar: 50 μm.

We did not detect any CD8-GFP signal in the female reproductive system (Figure 3A), while in the male reproductive system the signal distribution showed an interesting pattern. These data were confirmed with qPCR that showed a great difference between *sws* transcripts level in ovaries and testes (Figure 3B). The distribution of the signal in the testis was reminiscent of the mature cyst cell localization (Figure S1). In the proliferative apical tip of the testis there was no CD8-GFP signal, whereas near the needle-shaped spermatid nuclei there was a bright green contour (Figure 3C). The seminal vesicle epithelium was also CD8-GFP positive during the whole studied period of the fly life, even though the green signal in these cells was weak. Moreover, with age, it accumulated inside the seminal vesicle lumen (Figure 3D and Figure S2, Supplementary Videos S1 and S2). The epithelium of the anterior ejaculatory duct had reporter expression only in 2–3-day-old males, completely disappearing by the 15th day (Figure 3E and Figure S3). Throughout the first 75 days of imago life, we discovered the signal in some secondary cells of the accessory glands that take part in secretion of seminal fluid (Figure 3F and Figure S4). In addition, these data were supported with the nuclei fluorescence pattern observed in 5-day-old transgenic males of *sws-GAL4;UAS-NLS-DsRed* genotype (Figure S5).

### 3.2. Normal sws Expression Is Required for Organism Viability

**Figure 4:**
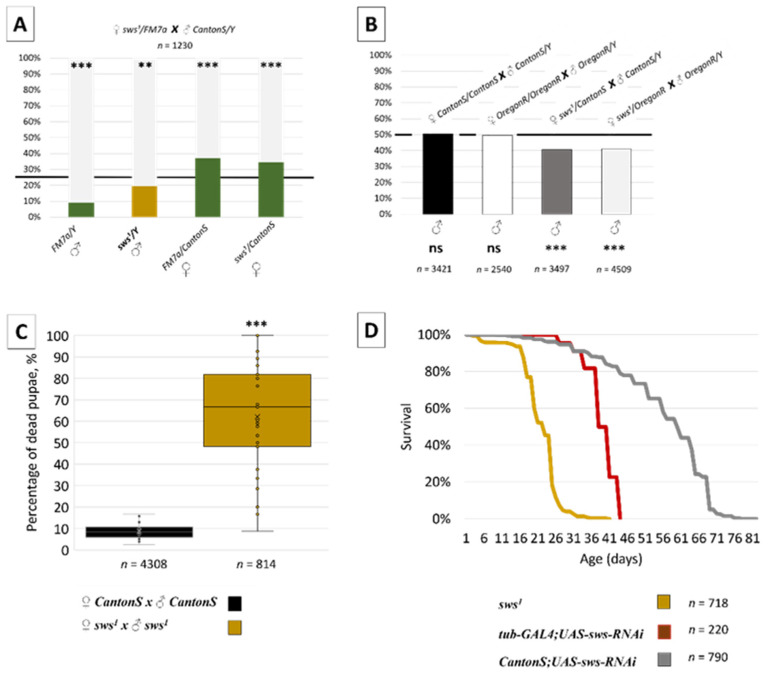
Lethality and survival analysis. (**A**) Percentage of different genotypic classes in the F1 progeny, obtained from crossing *sws^1^/FM7a* females with *CantonS* males. The total number of the first-generation imagoes is designated as *n*. A chi-squared statistical test was applied to test a hypothesis of unequal segregation in F1, ** *p* < 0.01, *** *p* < 0.001. Note that there are fewer mutant males than expected. (**B**) Percentage of the total male number in the F1 generation, obtained from different crossings. The total number of the first-generation imagoes is designated as *n*. A chi-squared statistical test was applied to test a hypothesis of unequal segregation in F1; *** *p* < 0.001; ns—no significant difference (*p* > 0.05). Note that there are fewer males in F1 than expected only in cases where *sws^1^* males are expected to be born. (**C**) Percentage of dead pupae in the F1 progeny, obtained from *CantonS* or *sws^1^* parents. The total number of the analysed first-generation pupae is designated as *n*. Mann–Whitney test; *** *p* < 0.001; the sample size is 30 examined vials. Note that there are much more dead pupae in the progeny of *sws^1^* mutant parents than of wild type *CantonS* parents. (**D**) Percentage of living imagoes with different genotypes depending on their age. The total number of counted imagoes is designated as *n* (100%).

Having proved the fact that *sws* promoter is active in several organs during different stages of *Drosophila melanogaster* development, we hypothesised that *sws* dysfunction could have general detrimental effects, affecting the fly viability. Therefore, we studied the survival of *sws^1^* mutants (*C7963853A*, Ser375*) that can synthesise only tremendously shortened, highly likely non-functional, SWS protein [9]. Since the *sws^1^* stock is extremely weak, it is maintained with the first chromosome balancer; for this purpose, *FM7a* is used in our lab. To obtain Cantonized *sws^1^* males, we crossed *sws^1^/FM7a* females with *CantonS* males and analysed phenotypic classes in F1 generation. We observed that the percentage of the F1 *sws^1^* males was significantly lower statistically than expected (Figure 4A). In the progeny of the normal (since the *sws^1^* mutation is recessive) heterozygous *sws^1^* females, we saw a similar phenomenon (Figure 4B). At least in part, this phenotype was prompted by pupal lethality since in the progeny of crossing a homozygous *sws^1^* female with a hemizygous *sws^1^* male we found an extremely high rate of pupal deaths (Figure 4C). Complete SWS function alteration in a whole organism is needed for this phenotype manifestation, and a pancellular or tissue-specific RNAi-induced knockdown is not enough for the pupal lethality induction (Figure S6).

In addition, the *sws* dysfunction influences the imago survival in the case of *sws^1^* mutants, and the partial SWS downregulation induced by the pancellular *sws* knockdown (*tub-GAL4;UAS-sws-RNAi*, Figure 4D) has a similar result. Therefore, the expression of normal *sws* in an organism is vital for, at least, pupae and imagoes, even though some *sws^1^* mutant pupae and flies are able to develop and survive.

### 3.3. Normal sws Expression Is Required for Reproduction

Taking into account the *sws* expression abundance in the male reproductive system, we hypothesised that dysfunction of SWS could affect the fly fitness not only via survival decline but also through reproductive function alterations.

**Figure 5:**
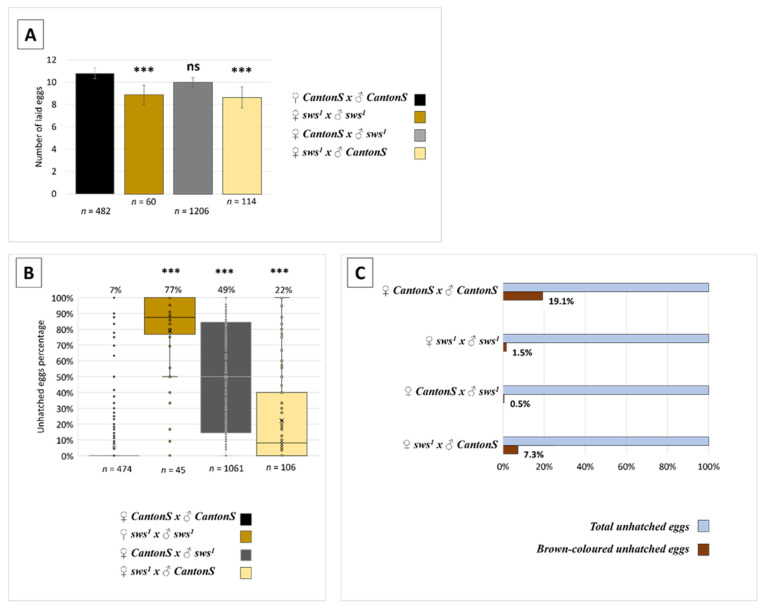
Analysis of fertility. (**A**) Female fecundity (number of eggs laid by 1 female per 24 h) in different crossings. Parents’ genotypes are written near the diagram. The sample size for each crossing is designated as *n*. The age of parents was equal, ranging from 3 to 10 days. Dunnett’s test; *** *p* < 0.001; ns—no significant difference (*p* > 0.05). Note that the *sws^1^* mutant mothers laid fewer eggs than the respective controls. (**B**) Percentage of unhatched eggs laid by a normal/mutant female crossed with a normal/mutant male after 48 h of incubation of the clutch. The mean is written above each bar. Parents’ genotypes are written under the diagram. The sample size for each crossing is designated as *n*. The age of parents was equal, ranging from 3 to 10 days. Steel test; *** *p* < 0.001. Note that there are much more unhatched eggs in the progeny of mutant parents, and this phenotype is partially copied when even one of the parents is mutant. (**C**) Percentage of brown eggs among the total number of unhatched eggs in the progeny of flies in different crossings (brown colour indicates the egg was fertilized). Note that in the progeny of a mutant parent there are fewer brown unhatched eggs.

We found that the *sws^1^* females lay fewer eggs than the controls (Figure 5A). These results suggest the normal *sws* expression to be required for female fecundity; however, this role is evidently minor.

Moreover, most of the eggs laid did not develop into larvae in the case of *sws^1^* mutant parents (Figure 5B). These eggs were morphologically similar to unfertilised eggs. The reduced hatchability of the eggs in the case of *sws^1^* mutants was caused by both father’s and mother’s genotypes (Figure 5B). It indicates that the normal *sws* function is required in females and males for their fertility. If a father was a mutant and a mother was a wild type (*CantonS*), the mean percentage of unhatched eggs was higher than in the reciprocal crossing, suggesting that fertility is affected stronger in the male organism due to *sws* dysfunction than in the female one (Figure 5B). We should note that in the case of a *sws^1^* mutant father, there were substantially fewer brown eggs than in the case of a *CantonS* father (Figure 5C). Eggs become brown if there is an embryo inside, but their development interrupts after gastrulation. This fact can potentially mean that the unhatched eggs were truly unfertilized. Furthermore, with aging, *sws^1^* mutant parents left less viable offspring. Since the age of 10 days, flies gave less progeny: there were few pupae in the first generation of the mutant parents, while the wild type flies left much more offspring (Figure S7A). Even though the 15-day-old mutant females, when crossed with the 15-day-old *CantonS* males, left many eggs that hatched, the 15-day-old *CantonS* females left few eggs that were able to hatch when crossed with the 15-day-old mutant males (Figure S7B). These data are a strong argument for the *sws* role in the *Drosophila* male reproduction control.

### 3.4. Dysfunction of sws Alters Male Behaviour

**Figure 6:**
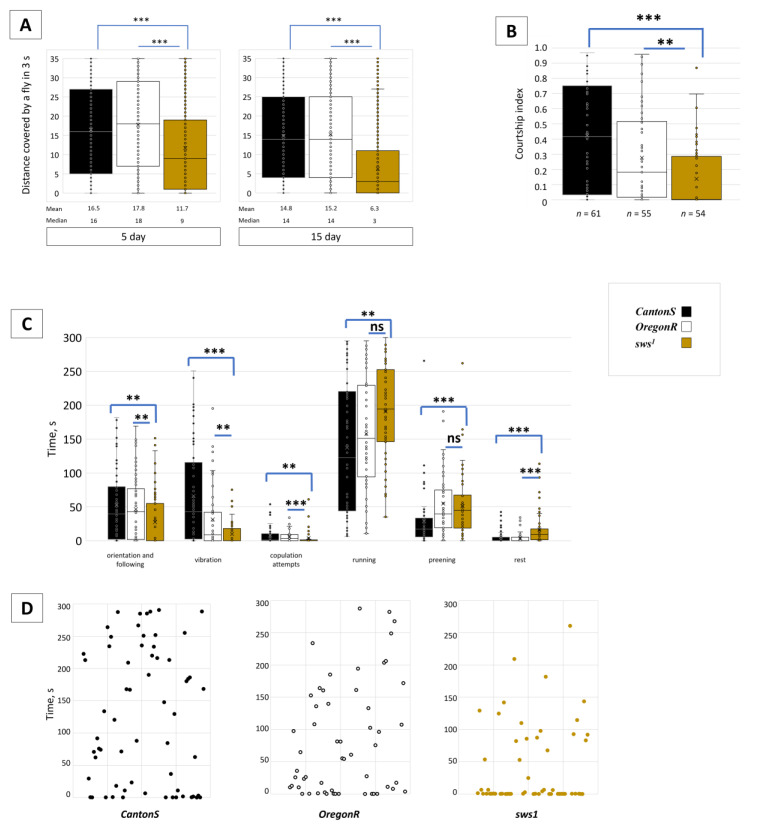
Behavioural analysis. (**A**) Locomotor activity of 5 and 15-day-old males; *n* = 2000. (**B**) Courtship index (the ratio of courtship time to the total time of behaviour examination) of 5-day-old males; the sample size is written under each bar. (**C**) Time spent for different types of male behaviour, of the total registration period of 300 s (when 4–6-day-old males were coupled with a mated *CantonS* female); the sample size is equal to (B). (**A**–**C**) Steel test; ** *p* < 0.01; *** *p* < 0.001; ns—no significant difference (*p* > 0.05). (**D**) Total time of sex behaviour (orientation, following, vibration, copulation) of individual males among the total 300 s registration time. Note that most of the *sws^1^* mutant males spend less time for courtship than the wild type *CantonS* and *OregonR* controls.

Since the fertility of *sws^1^* males was affected more severely than the female one, we thought about potential reasons for the role of *sws* in the male reproductive function. First of all, we analysed the imago behaviour of *sws^1^* male mutants, because it is shown that the *sws* function is necessary for neurons and glia in the central and peripheral nervous system [11,12,13]. Locomotor activity of the *sws^1^* males was lower, than that of the wild type *CantonS* and *OregonR* flies (Figure 6A). In addition, sexual behaviour was also affected in the *sws^1^* mutants whose time spent for courtship was reduced in comparison to the controls (Figure 6B). We noticed that all behavioural stages of courtship were performed by the *sws^1^* males less actively (Figure 6C). Even though several of them were able to court as long as the *CantonS* or *OregonR* males, most of the mutants did it only for a short time (Figure 6D).

**Figure 7:**
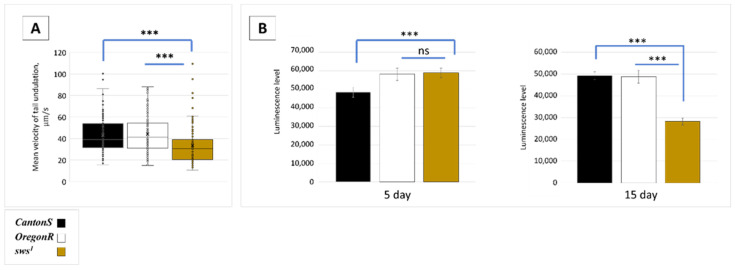
Spermatozoa analysis. (**A**) Mean velocity of a tail end undulation of a spermatozoid, extracted from a seminal vesicle, *n* = 111. Steel test, *** *p* < 0.001. Note that the performance of the *sws^1^* mutants is weaker than that of the controls. (**B**) The level of luminescence corresponding to the level of ATP extracted from testes and seminal vesicles of 5 and 15-day-old males. Dunnett’s test, *** *p* < 0.001, ns—no significant difference (*p* > 0.05), *n* = 100.

Moreover, there is another relation of SWS to male fertility. It is associated probably with *sws* expression in the male reproductive system. The evidence for it is the fact that spermatozoa from the seminal vesicles of the mutant males were less active since their mean velocity of a tail undulation is inferior to the control one (Figure 7A). These data give the basis for the reduced fertility of *sws^1^* mutant males who court weaker and whose sperm is not active enough for effective egg fertilisation. Therefore, the cumulative reason for this phenomenon is ablation of both (1) nervous system regulation of sexual behaviour and (2) spermatogenesis/sperm maturation/sperm competition.

Observing reduced locomotion of the *sws^1^* mutant spermatozoa, we proposed that it could be associated with diminished energy supply. Surprisingly, in 5-day-old mutants, the ATP level in testes and seminal vesicles was at the rate of the control. However, in the 15-day-old mutants, the level was lower than the control one (Figure 7B). This could mean that the primary disturbances of fertility in the young males may not be induced with the energy supply of spermatozoa.

### 3.5. Physiological Changes in Testes of sws^1^ Mutant Males

**Figure 8:**
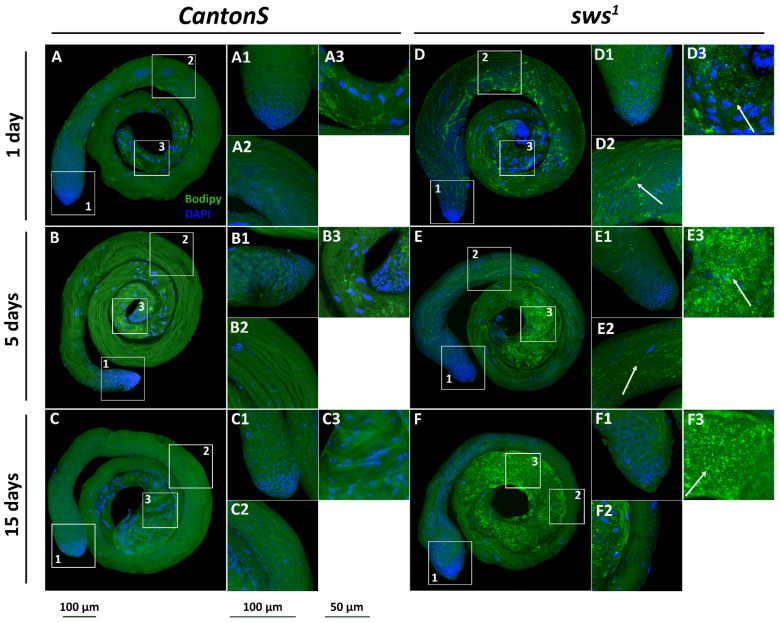
Lipid droplet analysis. 3D confocal stacks of testes stained with BODIPY 493/503 (green), showing the distribution of neutral lipids in *CantonS* (**A**–**C**) and *sws^1^* (**D**–**F**) males of different ages. Areas in white boxes were scanned additionally to obtain magnifications of (**1**) apical tip, (**2**) middle zone, and (**3**) proximal region. Lipid droplets are bright green dots (marked with arrows). Nuclei are stained with DAPI (blue). Scale bar is written under columns: 100 µm (whole images designated with letters; magnifications (**1**,**2**)), 50 µm (magnifications (**3**)). Note that there are few LDs in control testes, but LDs are abundant in *sws^1^* mutant testes.

Lipid metabolism has an essential role in male germ cell development and maturation, and its disturbance can result in lipid droplets (LDs) accumulation in somatic cells, compromising male fertility [55,56,57,58]. Considering the role of the SWS protein in lipid metabolism, we analysed lipid droplet distribution after staining testes with the BODIPY 493/503 dye. We found that the testes of *CantonS* males did not contain any distinct LDs, opposing to the 1-day-old *sws^1^* mutants that had LDs in, apparently, cyst cells near spermatids, but not near other sperm lineage cells. By the 15th day, LDs in the mutants changed their localization to the proximal end of the testis (Figure 8). We hypothesised that LDs accumulation could be provoked by oxidative stress as it was previously shown in several studies [13,59,60]. Nevertheless, the reactive oxygen species level in the mutant testes did not differ from the control ones (Figure S8), so we propose that in this case oxidative stress is not the reason of LDs overrepresentation.

## 4. Discussion

In the current study, we have analysed the expression profile of the PC (lyso)phospholipase gene *sws* in *Drosophila melanogaster* ontogenesis. Using transgenic *sws-GAL4;UAS-CD8-GFP* flies, we have described the spatiotemporal pattern of the *sws* promoter activity and elucidated organs in which the *sws* gene is expressed. The reporter signal appeared in the 16-stage embryo in the salivary glands and in the 17-stage embryo in the fat body cells and then remained in these organs during larval stages. In the larvae, we also found the signal in the digestive system and Malpighian tubes. At the imago stage, the signal was detected in the adipocytes, Malpighian tubes, and midgut as well. These data confirm the previous findings of transcriptomic analyses available at FlyBase and FlyAtlas [44,45].

Since *sws* is expressed in a fruit fly at different developmental stages, we analysed whether its function is necessary for an organism survival. To do this, we studied *sws^1^* mutants that have no functional protein due to the nonsense mutation [9]. These mutants demonstrated increased lethality at the imago stage and previous stages, e.g., the pupal one. The ubiquitous knockdown of *sws* in *tub-GAL4;UAS-sws-RNAi* specimens also induced imago lethality, though it happened later if compared with the *sws^1^* mutants. Importantly, while the neuronal *sws* knockdown induced reduction of lifespan in imago [13], it did not influence the flies’ survival during their earlier developmental stages and neither did the panglial *sws* knockdown despite the importance of *sws* in larval peripheral glia [12,61]. These findings suggest that residual SWS activity in the case of a knockdown could partially rescue pupae and imagoes from premature death, even though some *sws^1^* mutants with total *sws* dysfunction can survive for some time. Moreover, it seems that SWS activity alteration in the nervous system (which has been studied in depth [9,10,11,12,13,61]) is not the reason for the pupal mortality.

We did not find any reporter signal in the female reproductive organs; however, the transcriptomic data [44,45] and our PCR analysis revealed *sws* transcripts in ovaries. This contradiction could be explained by the detection system used. Probably, the activation of *sws* promoter was not enough to induce CD8-GFP production at levels appropriate for detection with a confocal microscope. Nevertheless, the level of *sws* expression in ovaries is much lower than in testes. We found that the mutant females laid fewer eggs than the wild type *CantonS* or *OregonR* females, and among the laid eggs, about a quarter could not develop into larvae, suggesting that *sws* disruption in females affects fecundity and fertility.

In addition, we found a very interesting pattern of *sws* expression in the male reproductive system. We propose that *sws* is expressed in cyst cells of testis. The expression was more pronounced in the middle and the proximal end of the testes, whereas there was no CD8-GFP signal in the proliferative zone where mitotic and meiotic divisions of sperm cell predecessors occur. With aging, the signal intensity in the testes was decreasing. In addition, *sws* is expressed in epithelial cells of seminal vesicles, ejaculatory duct, and accessory glands. These cells are important for the sperm development and maturation, and they participate in the secretion of some factors into the sperm fluid [62,63,64,65]. Therefore, SWS lysophospholipase may somehow regulate normal secretion there. It is noteworthy that the murine SWS orthologue was previously proposed to regulate secretion in the cerebellar granule neurons [7], while PNPLA6 inhibition in the gonadotrope cell line resulted in the prevention of exo/endocytosis [25]. Hence, SWS may be important in somatic cells of the fly reproductive system to assist their physiological role in the organism through soma-germ intercellular communication and sperm fluid generation.

The most intriguing phenotype which we have revealed in the current study was associated with the male fertility that was severely disturbed under *sws^1^* mutation. The mutant males had a reduced locomotor behaviour and courtship activity, suggesting that they might fertilise females worse than the wild type flies. It is interesting to add that SWS function might have a potential role in the courtship control since its partner, the catalytic subunit of a protein kinase A, namely, PKA-C3, is important for the male copulation behaviour [66]. The reduction of copulation attempts was induced by the downregulation of PKA-C3 in two pairs of interneurons [66]. SWS is shown to be a regulatory subunit that can recruit PKA-C3 and inhibit it [67]. Therefore, behavioural deficits induced by the absence of normal SWS because of the *sws^1^* mutation can be mediated by PKA-C3 activity changes.

As a result of the *sws^1^* mutation, slightly fewer than half of the eggs in the progeny of the mutant males seemed to be unfertilized. It could be also due to the mutants’ spermatozoa that were less active than those of the wild type ones. The phenotype worsened with the male age, and the 15-day-old mutant males became almost sterile. Interestingly, the levels of ATP extracted from the testes and seminal vesicles of the aged mutant males were lower, than those in the wild type flies.

The male infertility phenotype may be associated with some consequences that occur in cyst cells which have been shown to express *sws*. These cells develop in tight interdependence with germ line and regulate spermatogonial proliferation, spermatocyte survival, spermatid elongation, and sperm maturation [68]. Various coordinated interactions of *Drosophila* germ and soma line cells that result in codifferentiation have been studied in detail [69,70,71,72]. Cyst cells appeared to be sensitive to lipid metabolism, and their survival depends on fatty acid utilization [73,74]. When it is aborted, fatty acids are stored in lipid droplets that are accumulated in cyst cells [74]. It was surprising that in the case of *sws^1^* mutants, LDs were found in the testes, presumably in the cyst cells. Such phenotype may be a marker of mitochondrial deterioration [74]. However, in some cases LDs were supposed to perform a protective role for lipids against peroxidation [13,59,60]. In mammalian analogues of cyst cells, namely, Sertoli cells, that build a niche and support germ proliferation and differentiation, LD accumulation appears as a result of phagocytosis of apoptotic spermatogenic cells [75], intoxication [76], and prolonged hyperthermic stress [77]. In addition, LDs seem to supply spermatogenesis via lipophagy and lipid oxidation [78,79,80].

In this way, even though the *sws* gene is known to encode lysophospholipase, the specific mechanism of its action in the male reproductive system demands further elaborate investigation. We propose that in the case of *sws^1^* mutants, fatty acid release from phospholipids is diminished. The lack of fatty acid liberation may alter intracellular signalling since many unsaturated fatty acids and their derivatives are versatile messengers that regulate inflammation, calcium storage, blood pressure, the neurotransmitter system as well as membrane fluidity [81,82,83,84,85]. A lot of studies demonstrate that polyunsaturated fatty acid composition in testis germ and soma is important for spermatogenesis, sperm maturation and competition [86,87,88,89,90,91,92,93,94,95,96,97]. Importantly, it is shown that polyunsaturated PC and lysoPC levels rise under *sws* malfunction [15]. It may provoke additional negative effects since lysoPC was shown to be enriched in spermatozoa with deteriorated membranes [98]. Increased lysoPC content was also found in obese men, and it correlated with reduced sperm motility [99,100]. It is of note that many diseases are associated with elevated levels of this lipid [101]. At the same time, lipolysis with lysoPC production is necessary for proper spermatozoa maturation and capacitation [102,103]. These data mean that accurate regulation of lysoPC level in a cell is important, rather vital, because of biophysical properties of this molecule that acts as a detergent in biological membranes and a messenger for signalling [104]. Furthermore, PC metabolism should be regulated since its by-products, lysoPC and either saturated or unsaturated fatty acids, have a wide variety of functions [105]. SWS is a regulator of such metabolism, and we have determined that its function is very important in the male reproductive system of the fruit fly.

## 5. Conclusions

In the current study, we have demonstrated that the SWS lysophospholipase gene is expressed in several types of somatic cells in the *Drosophila melanogaster* male reproductive system, and it is required for male fertility, suggesting that the biochemical activity of this enzyme is necessary for normal sperm formation and function. Therefore, not only the nervous system but also the reproductive system of the fruit fly depends on SWS function. Although we have shown that *sws* is expressed in various cells, we currently do not know whether it is vital for them. If some of these cells can survive despite SWS dysfunction, it is important to know why it is possible. Such studies might have some significant implications in finding out the mechanisms of cell survival maintenance, especially because of the medical significance, since mutations in the human *sws* orthologue *PNPLA6*, or organophosphate poisoning-induced inhibition of PNPLA6 protein, have neurodegenerative effects [106].

All things considered, *Drosophila melanogaster* fitness depends on the normal functioning of the *sws* gene. The latter pleiotropically regulates survival of specimens during several developmental stages and fertility of both sexes, and it also has a minor influence on female fecundity.

## Data Availability

The data presented in this study are available in the article and Supplementary Materials.

## Supplementary Materials

The following are available online at https://www.mdpi.com/article/10.3390/insects13010014/s1. Figure S1: Cells with GFP (green) expression in the testis of imago with *sws-GAL4;UAS-CD8-GFP* genotype. Figure S2: GFP (green) in the seminal vesicle of imago with *sws-GAL4;UAS-CD8-GFP* genotype. Figure S3: Cells with GFP (green) expression in the anterior ejaculatory duct of imago with *sws-GAL4;UAS-CD8-GFP* genotype. Figure S4: Cells with GFP (green) expression in the accessory gland of imago with *sws-GAL4;UAS-CD8-GFP* genotype. Figure S5: Nuclei with NLS-DsRed (red) expression in the male reproductive system of 5-day-old imago with *sws-GAL4;UAS-NLS-DsRed* genotype. Figure S6: Segregation analysis of *sws* knockdowns. Figure S7: Number of pupae and eggs in the progeny of flies with different genotype depending on the age of the parents. Figure S8: Fluorescence level, corresponding to the reactive oxygen species level in testes and seminal vesicles of 5 and 15-day-old males. Supplementary Video S1: Confocal stack of the seminal vesicle of the 5-day-old male with *sws-GAL4;UAS-CD8-GFP* genotype (green—CD8-GFP, blue—nuclei stained with DAPI; above the seminal vesicle, there is the proximal part of the testis). Supplementary Video S2: Confocal stack of the seminal vesicle of the 30-day-old male with *sws-GAL4;UAS-CD8-GFP* genotype (green—CD8-GFP, blue—nuclei stained with DAPI).
