# Supplementary material for: Drosophila Lysophospholipase Gene swiss cheese Is Required for Survival and Reproduction
Source: Insects. 2021 Dec 22;13(1):14. doi: 10.3390/insects13010014 (PMC8781823; doi:10.3390/insects13010014)
Supplement: Supplementary file 1 [file insects-13-00014-s001.zip › insects-1499843-proofed-s.pdf]

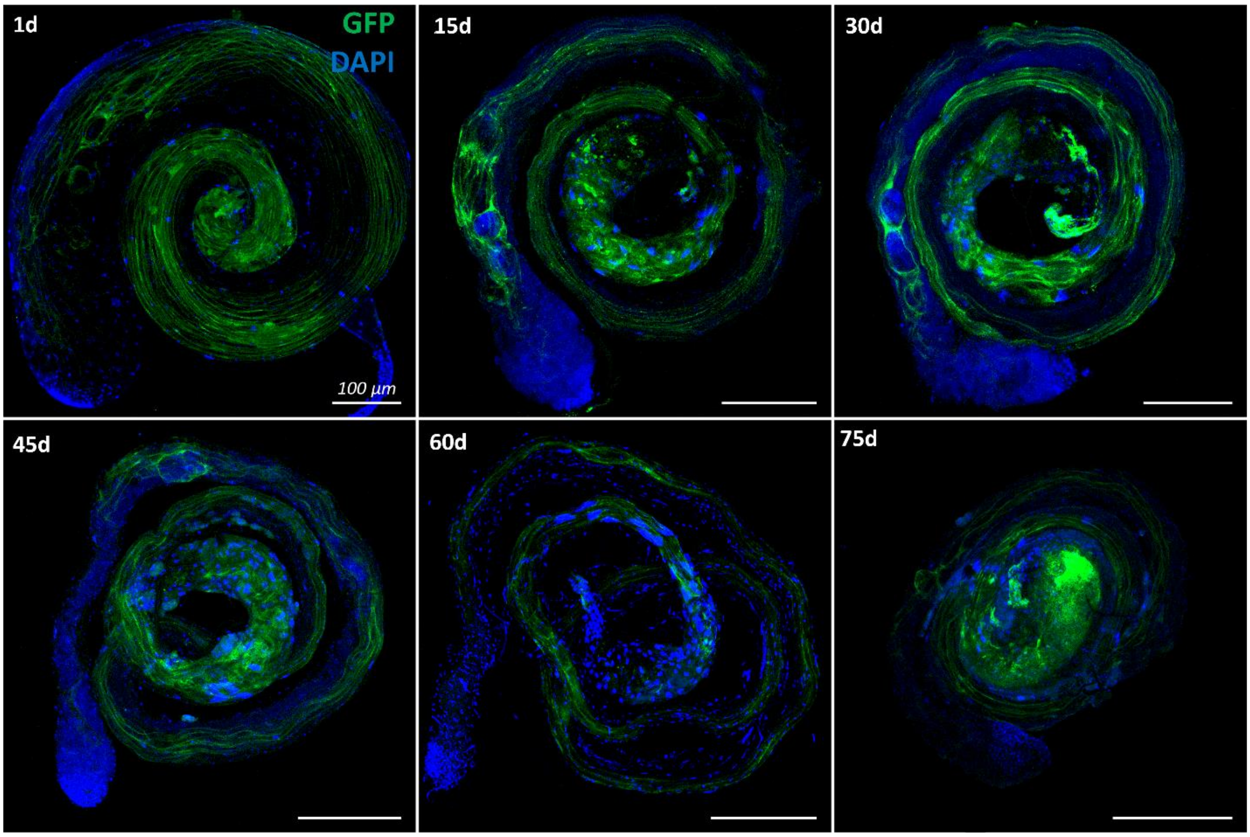


**Figure S1.** The cells with GFP (green) expression in the testis of imago with *sws-GAL4;UAS-CD8-GFP* genotype. Nuclei are stained with DAPI (blue). In the left upper corner of each photograph the age of imago (days) is written. Scale bar: 100 µm. The GFP signal pattern is reminiscent of mature cysts localization in the testis.

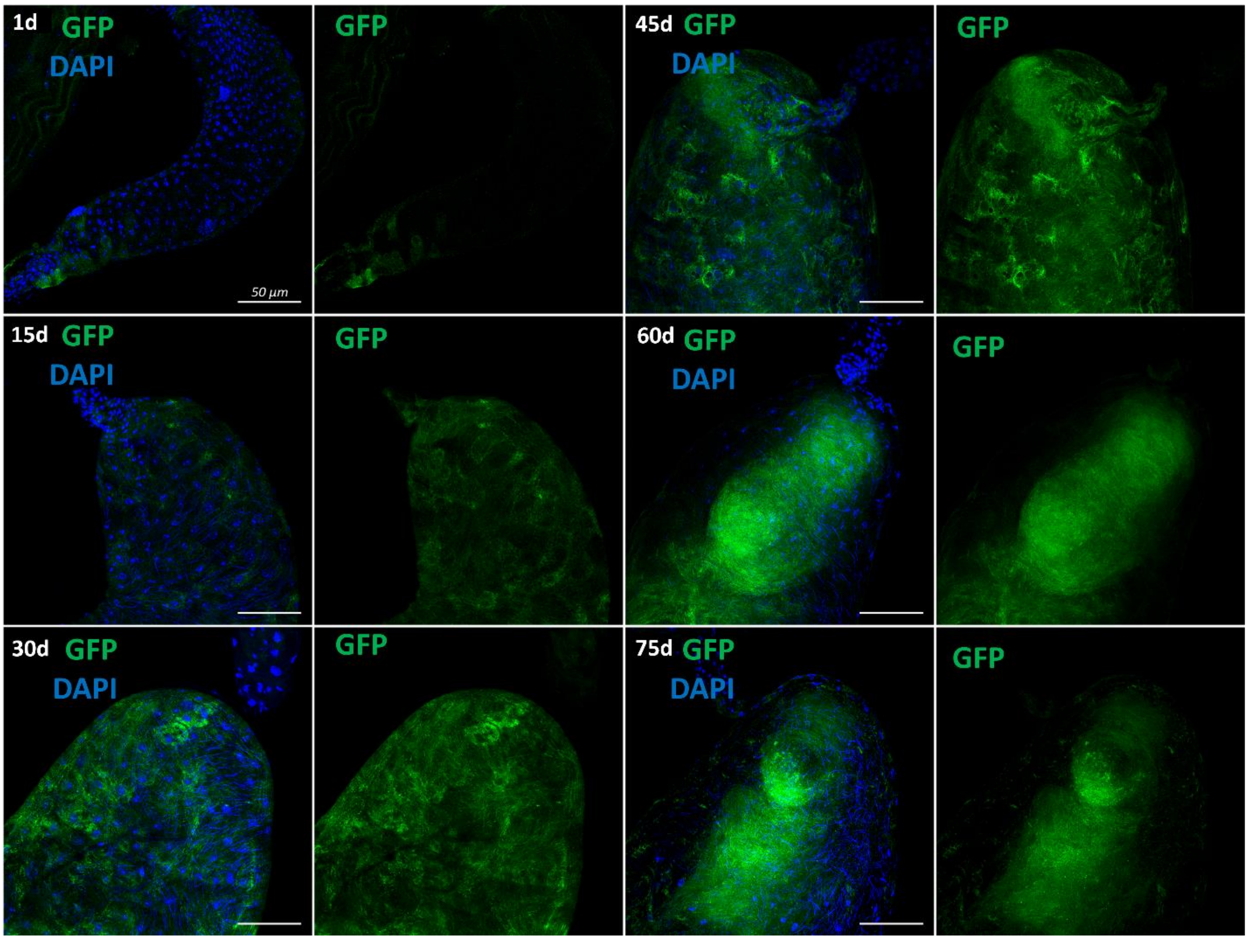


**Figure S2.** GFP (green) in the seminal vesicle of imago with *sws-GAL4;UAS-CD8-GFP* genotype. Nuclei are stained with DAPI (blue). In the left upper corner of each photograph the age of imago (days) is written. Scale bar: 50 µm. The GFP signal is weak and is found in the epithelial layer. The signal is absent in the lumen of the seminal vesicle in young flies (before day 15), but then accumulates inside the seminal vesicle with age.

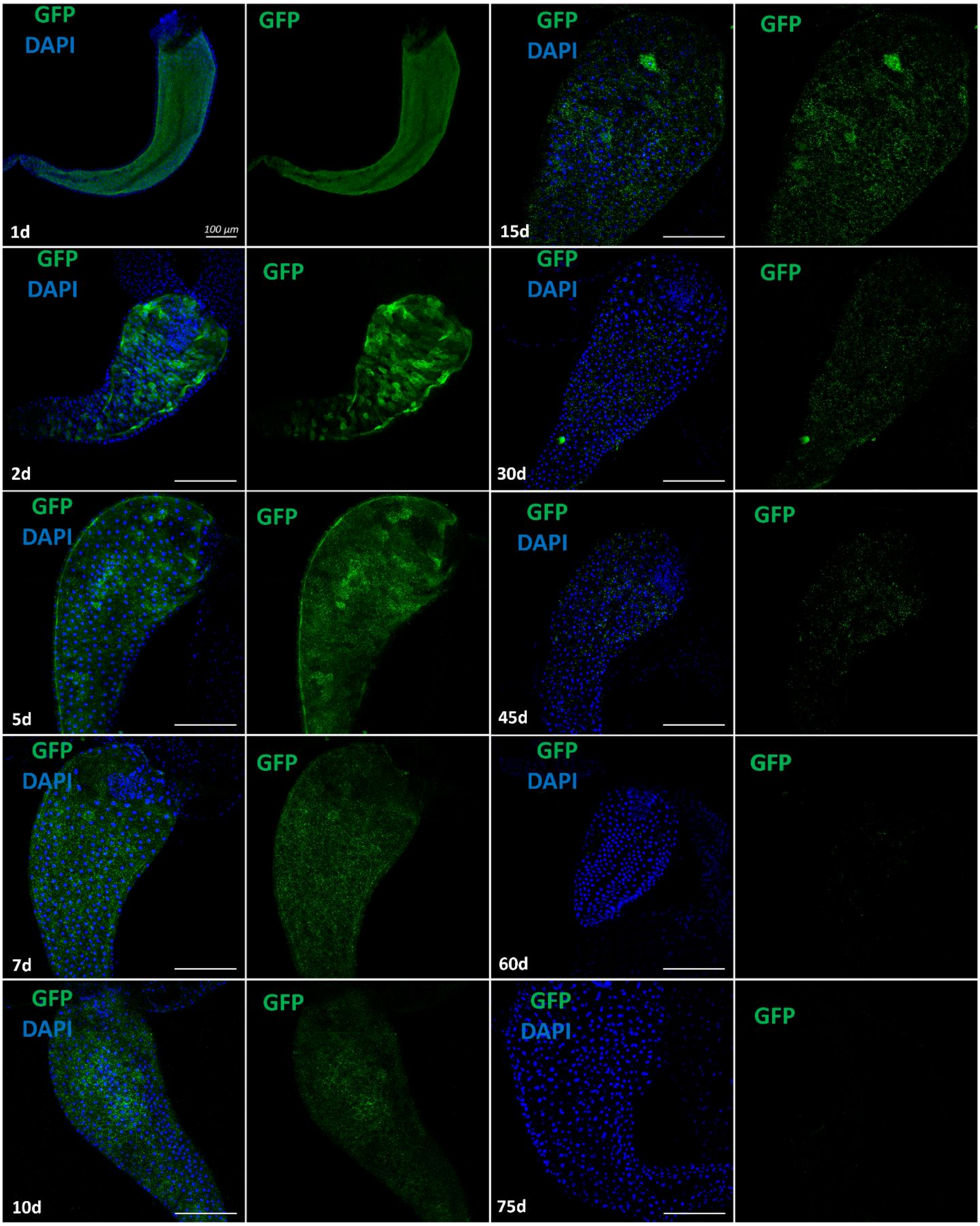


**Figure S3.** The cells with GFP (green) expression in the anterior ejaculatory duct of imago with *sws-GAL4;UAS-CD8-GFP* genotype. Nuclei are stained with DAPI (blue). In the left down corner of each photograph the age of imago (days) is written. Scale bar: 100 µm. The GFP signal is abundant in the epithelial layer. After the second day the GFP signal gradually disappears.

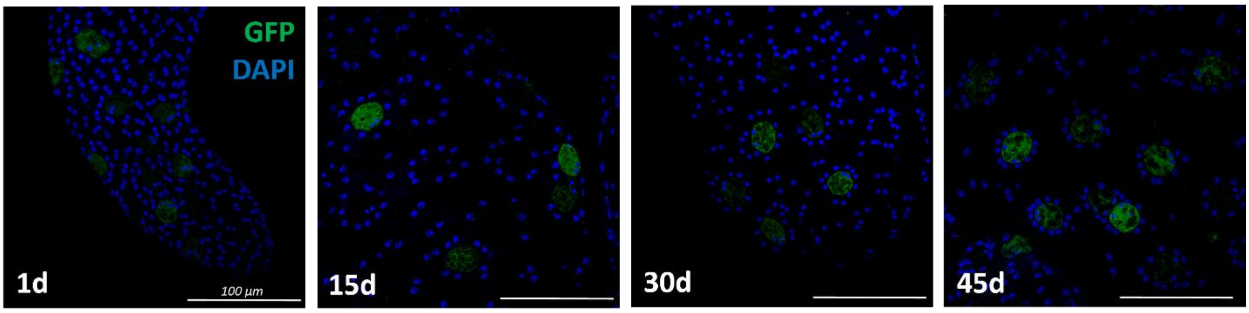


**Figure S4.** The cells with GFP (green) expression in the accessory gland of imago with *sws-GAL4;UAS-CD8-GFP* genotype. Nuclei are stained with DAPI (blue). In the left down corner of each photograph the age of imago (days) is written. Scale bar: 100 µm. The GFP signal is found in the large secondary cells of the epithelium layer of the accessory gland. It is visible that not all secondary cells are GFP-positive.

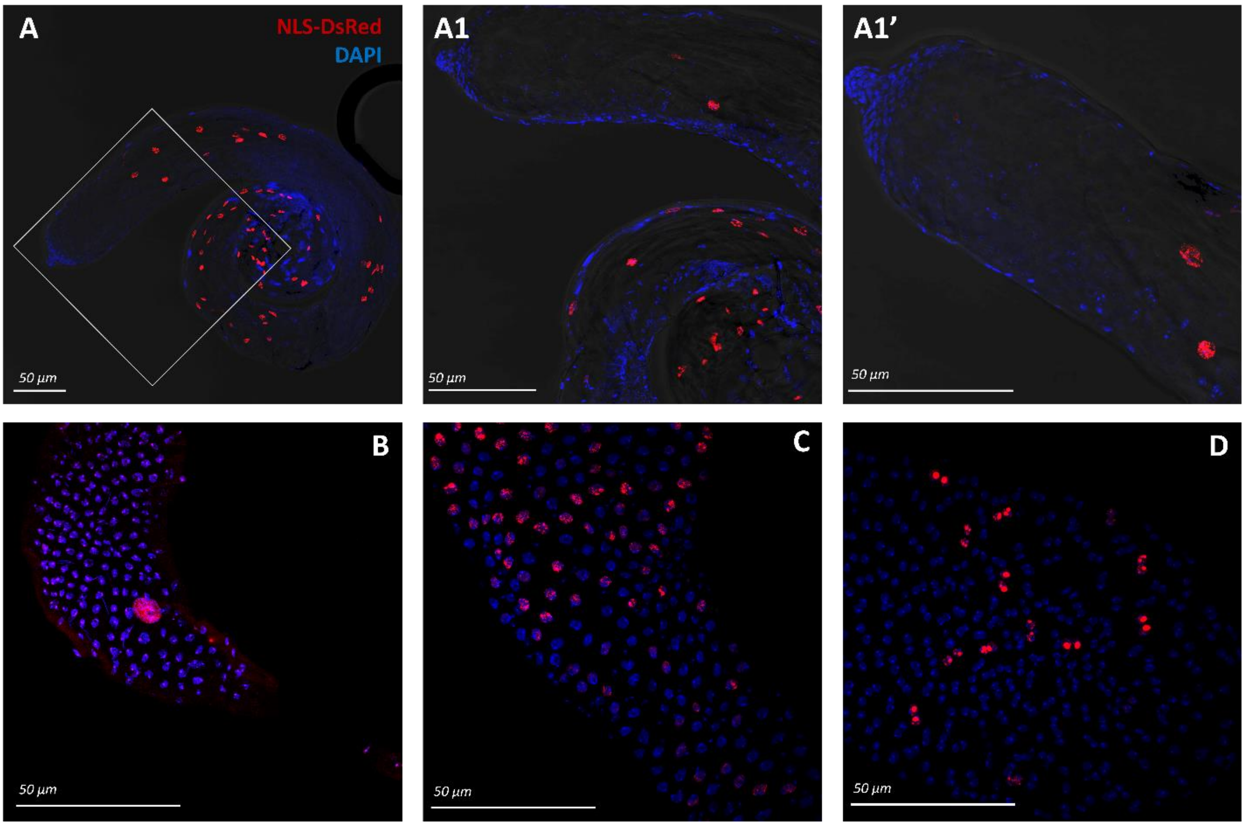


**Figure S5.** The nuclei with NLS-DsRed (red) expression in the male reproductive system of 5-day-old imago with *sws-GAL4;UAS-NLS-DsRed* genotype. Confocal stacks of (**A**) testis with 2X (**A1**) and 3X (**A1’**) magnifications, (**B**) seminal vesicle, (**C**) anterior ejaculatory duct, (**D**) accessory gland. Nuclei are stained with DAPI (blue). Scale bar: 50 µm. Note, that inside the testis there is only one type of red nuclei (presumably, of cyst cells). Other types of nuclei in the testis (in the apical tip, in germ cells, epithelium, muscles) do not show any red fluorescence (**A1’**). The signal is very weak in the seminal vesicle epithelium nuclei, while the big round nucleus of the other cell type (morphologically corresponding to the pigment sheath cell) has the brighter signal (**B**). In the anterior ejaculatory duct epithelium some nuclei has not the red signal (**C**). In the accessory gland only secondary cells’ nuclei (but not all) has the red signal (**D**).

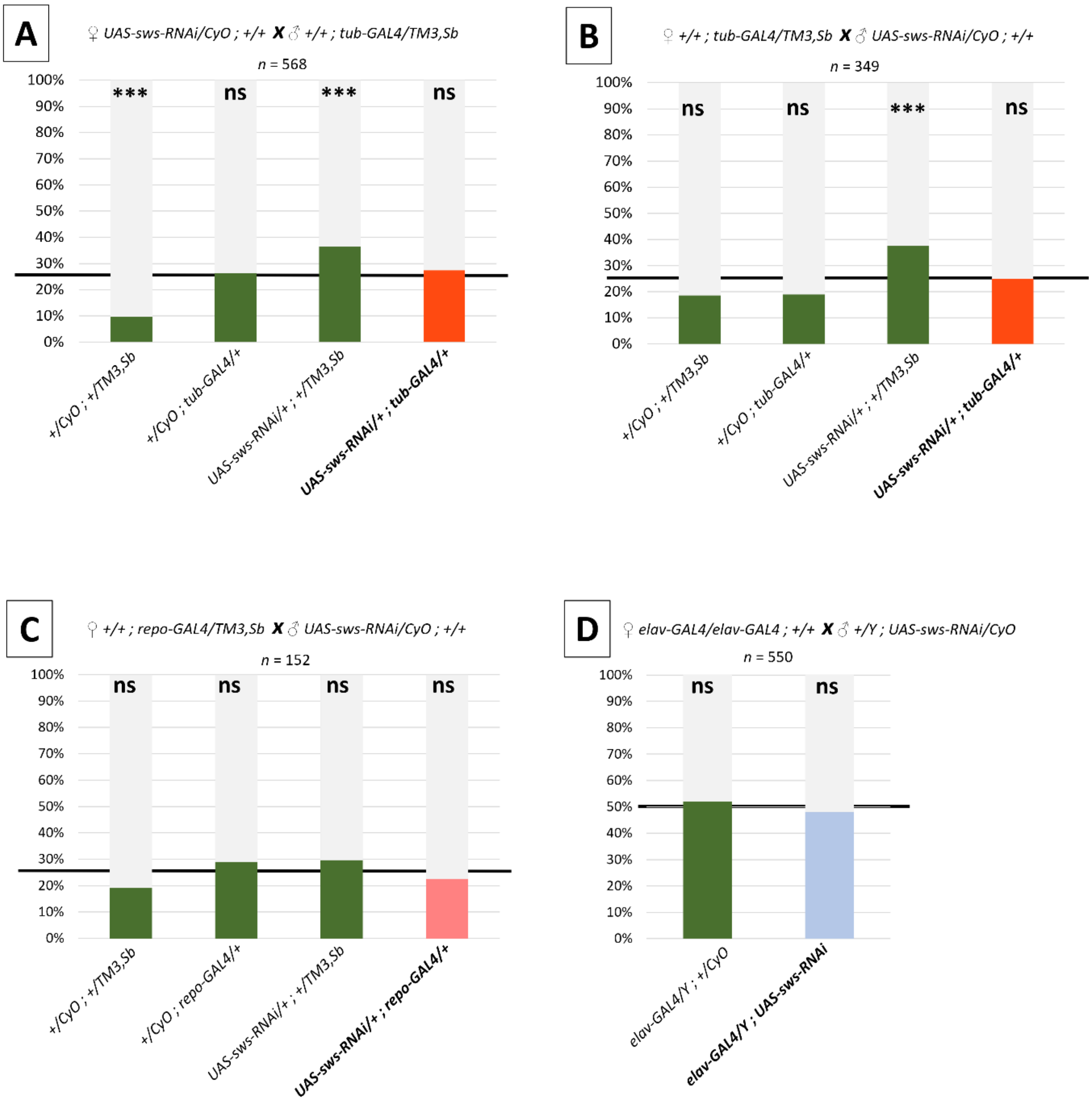


**Figure S6.** Segregation analysis of *sws* knockdowns. (**A**,**B**) The percentage of different genotypic classes in F1 progeny which includes pancellular *sws* knockdown flies (*tub-GAL4;UAS-sws-RNAi*), obtained from two reciprocal crossings. (**C**) The percentage of different genotypic classes in F1 progeny which includes panglial *sws* knockdown flies (*repo-GAL4;UAS-sws-RNAi*). (**D**) The percentage of different genotypic classes in F1 progeny which includes neuronal *sws* knockdown flies (*elav-GAL4;UAS-sws-RNAi*). (A–D) Only males were counted. The total number of the first-generation males is designated as *n*. Chi-squared statistical test was applied to test a hypothesis of unequal segregation in F1, *** $p < 0.001$, ns – no significant difference. Note, that the percentage of all these knockdowns do not significantly differ from the expected segregation rate.

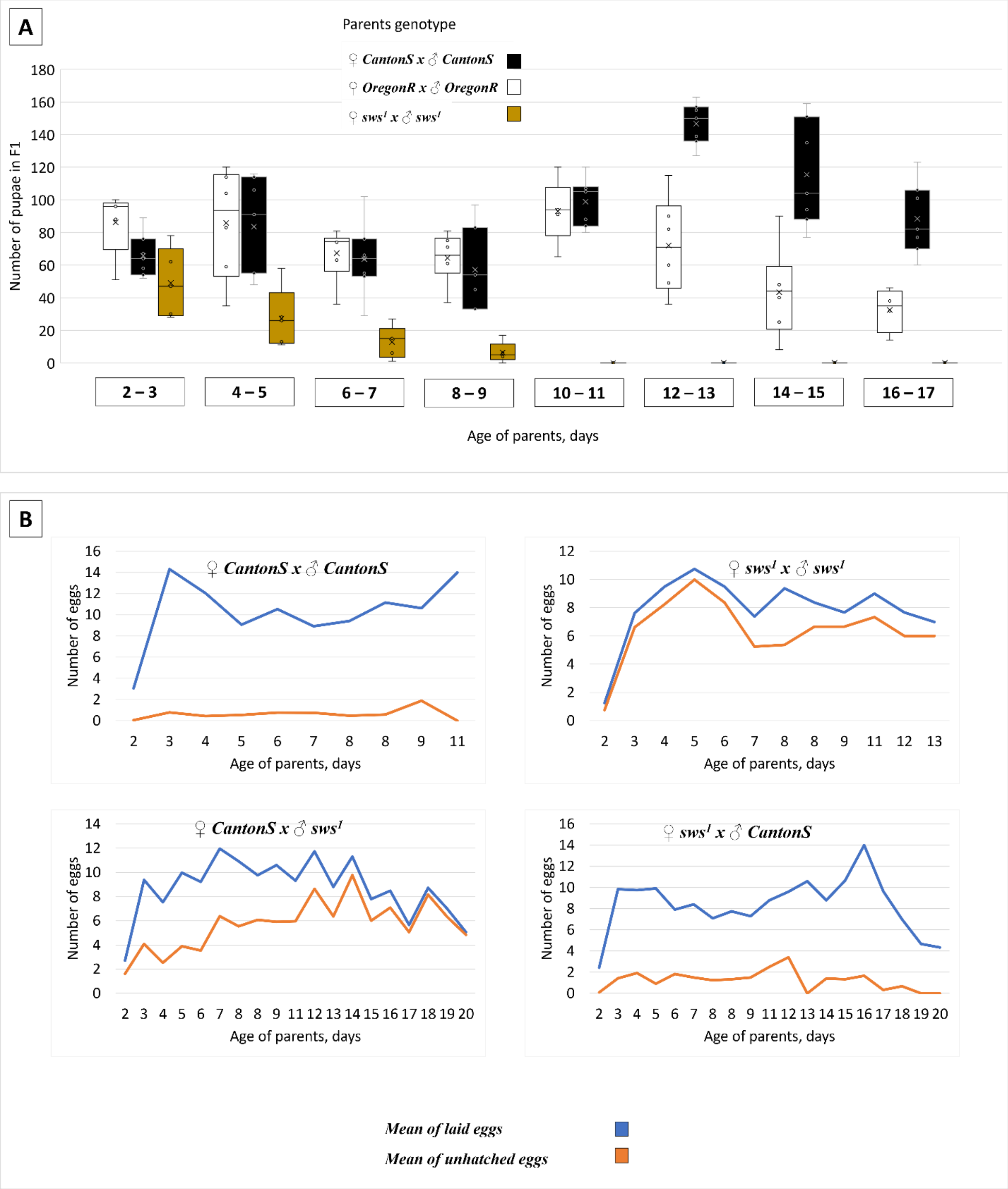


**Figure S7.** Number of pupae and eggs in the progeny of flies with different genotype in dependence of the age of the parents. (**A**) The number of pupae observed after 48 h crossing of 5 females and 5 males. The age of females was the same as the age of males in each crossing. (**B**) The mean number of eggs laid by one female for 24 h and the mean number of eggs stayed unhatched after 48 h of incubation. The parents were coupled together for all the period of the experiment. The sample size for every age point was more than 10 individual crossings. Note that in all cases with the mutant *$sws^1$* father the number of hatched eggs and pupae approximates to zero after the 10th day.

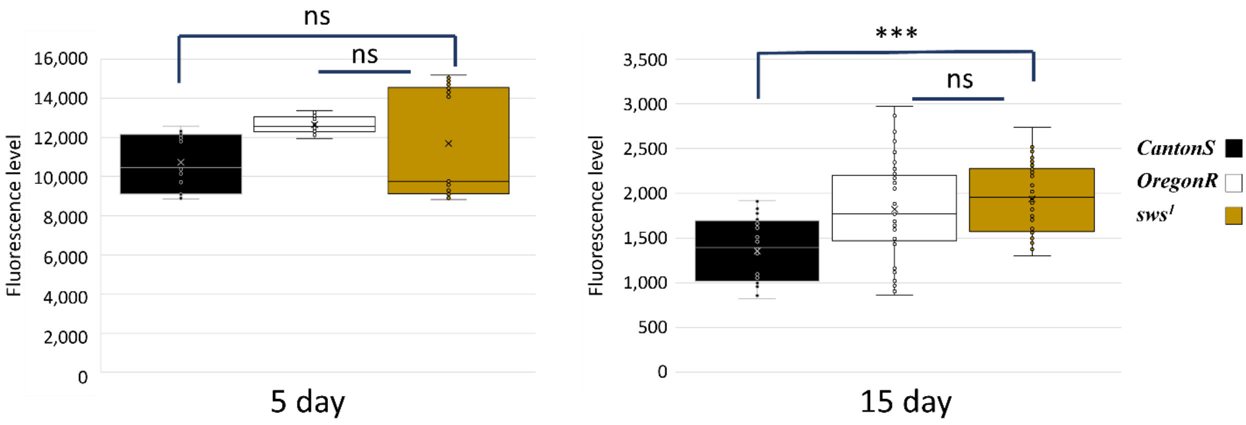


**Figure S8.** Fluorescence level, corresponding to the reactive oxygen species level in testes and seminal vesicles of 5 and 15-day-old males. Steel test, *** $p < 0.001$, ns – no significant difference, $n = 9$. There is no elevation of ROS in testes of *sws¹* males in comparison to both controls.
